# Effect of Long-Chain Fatty Acids on the Binding of Triflupromazine to Human Serum Albumin: A Spectrophotometric Study

**DOI:** 10.3797/scipharm.1310-23

**Published:** 2013-12-28

**Authors:** Keisuke Kitamura, Shigehiko Takegami, Rumi Tanaka, Ahmed Ahmed Omran, Tatsuya Kitade

**Affiliations:** 1 Kyoto Pharmaceutical University, 5 Nakauchicho, Misasagi, Yamashina-ku, Kyoto 607-8414, Japan.; 2 Chemistry Department, Faculty of Science, Al-Azhar University, Assiut 71524, Egypt.; 3 Chemistry Department, Faculty of Science, King Khalid University, Abha 9004, Saudi Arabia.

**Keywords:** HSA, Triflupromazine, Drug Protein Binding, Long-Chain Fatty Acid, Second-Derivative Spectrophotometry

## Abstract

Human serum albumin (HSA) in the blood binds long-chain fatty acids (LCFAs), and the number of bound LCFAs varies from 1 to 7 depending on the physical condition of the body. In this study, the influence of LCFA-HSA binding on drug-HSA binding was studied using triflupromazine (TFZ), a psychotropic phenothiazine drug, in a buffer (0.1 M NaCl, pH 7.40, 37°C) by a second-derivative spectrophotometric method which can suppress the residual background signal effects of HSA observed in the absorption spectra. The examined LCFAs were caprylic acid (CPA), lauric acid (LRA), oleic acid (OLA), and linoleic acid (LNA), respectively. Using the derivative intensity change of TFZ induced by the addition of HSA containing LCFA, the binding mode of TFZ was predicted to be a partition-like nonspecific binding. The binding constant (*K* M^−1^) showed an increase according to the LCFA content in HSA for LRA, OLA, and LNA up to an LCFA/HSA molar ratio of 3–4. However, at higher ratios the *K* value decreased, i.e. for OLA and LNA, at an LCFA/HSA ratio of 6–7, the *K* value decreased to 40% of the value for HSA alone. In contrast, CPA, having the shortest chain length (8 carbons) among the studied LCFAs, induced a 20% decrease in the *K* value regardless of its content in HSA. Since the pharmacological activity of a drug is closely related to the unbound drug concentration in the blood, the results of the present study are pharmaco-kinetically, pharmacologically, and clinically very important.

## Introduction

Human serum albumin (HSA), the most abundant protein in the blood (ca. 0.6 mM), has a strong ability to bind a large variety of endogenous and exogenous substances. Thus, most administered drugs are bound to HSA and transported in the bound state in the circulating blood [1–3]. Since only unbound free drugs in the blood can be transferred into body tissues, the pharmacological activity of a drug is closely related to the unbound drug concentration in the blood, which is in turn dominated by its binding to HSA [1–3].

Among important endogenous substances, long-chain fatty acids (LCFAs) have a strong tendency to bind to HSA, and the number of LCFAs bound to HSA is dependent on the physical conditions of the human body. Ordinarily, LCFA content in HSA is 1–2 molecules/one HSA molecule, however, upon fasting, after hard exercise, or in subjects with diabetes, 6–7 molecules of LCFAs bind to HSA [1, 4–6]. Therefore, if the LCFA-HSA binding affects the drug-HSA binding, this could alter the unbound concentration of the drug in the blood [1, 3] in a manner dependent on the LCFA content in HSA, and thus dependent on the physical condition of the body.

**Fig. 1:**
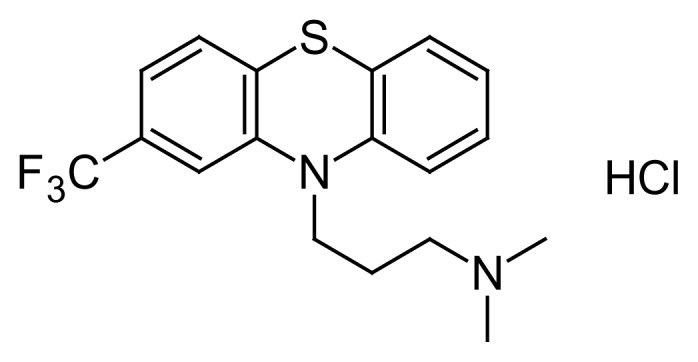
Structure of TFZ

Our previous ^19^F nuclear magnetic resonance (NMR) study found that a widely prescribed psychotropic phenothiazine drug, triflupromazine (TFZ, Fig. 1), binds to HSA and bovine serum albumin (BSA) with two different binding modes, i.e., specific binding to Sudlow’s Site II and nonspecific binding [7]. The ^19^F NMR study also revealed that oleic acid (OLA), one of the LCFAs, displaces the Site II-bound TFZ [7], showing that the binding of LCFAs affects the binding of TFZ to HSA. Therefore, a quantitative investigation into the effects of LCFAs on the binding of TFZ to HSA was required.

To determine the association constants of drugs to biological macromolecules such as BSA [8–11], liposomes [12–14], and lipid-emulsions [15] in buffer solutions, we have usually employed a second-derivative spectrophotometric method [16–19], since it can entirely eliminate the effects of background signals caused by the signals of macromolecules to offer a flat and zero-level baseline. In the previous studies [8, 9] to examine the phenothiazine drug-BSA binding, the binding constant was obtained by applying a simple partition-like nonspecific binding model, though the ^19^F NMR results showed that the TFZ-BSA binding involves specific Site II binding. This is because the ^19^F NMR signal intensity of the TFZ bound to Site II was less than one quarter of that bound nonspecifically, which made it possible to approximate the TFZ-BSA binding by nonspecific binding [7]. However, in the case of HSA, because the signal intensity of TFZ bound to Site II was larger than that in BSA, it would have been difficult to employ a simple partition-like nonspecific model as in the case of BSA. Conversely, in the presence of Cl^−^ at the physiological concentration of 0.1 M, the TFZ signal bound to Site II almost disappeared due to the displacement by Cl^−^ ions at Site II [7]. This suggests the possibility of applying the nonspecific binding model for evaluation of the effect of LCFAs on the TFZ-HSA binding as used for BSA [8, 9]. Thus, we quantitatively investigated the effect of LCFAs on the binding of TFZ to HSA in the presence of a physiological concentration of 0.1 M Cl^−^ using four types of LCFAs, caprylic acid (CPA), lauric acid (LRA), OLA, and linoleic acid (LNA), employing the second-derivative spectrophotometric method in this study.

## Experimental

### Chemicals

TFZ hydrochloride, HSA (essentially fatty acid-free), and sodium salts of CPA (8:0)*, LRA (12:0)*, OLA (18:1)*, and LNA (18:2)* were purchased from Sigma-Aldrich (St. Louis, MO, USA). Analytical reagent-grade sodium chloride was purchased from MERCK (Whitehouse Station, NJ, USA). All reagents were used without further purification. Stock solutions of TFZ (200 μM) and HSA (150 μM) were prepared in 0.1 M NaCl sodium phosphate buffer (0.05 M, pH 7.40). The exact concentration of HSA in a stock solution was determined by UV absorption using the absorptivity value at 279 nm, E_1cm_^1%^ = 5.31, and the molecular weight of 66.4 KDa [1]. Each LCFA stock solution was 6.5 mM LCFA sodium salt aqueous solution.

*Numbers in the parentheses denote the number of carbons and the number of unsaturated bonds contained in each LCFA, respectively.

### Preparation of Sample Solutions

LCFA-bound HSA buffer solution (LCFA-HSA stock solution) was prepared by mixing adequate amounts of LCFA and HSA stock solutions to attain a required LCFA/HSA molar ratio of 0 to 7. To a 5-mL volumetric flask, 0.5 mL of the TFZ stock solution and an aliquot of the LCFA-HSA stock solution sufficient to yield an HSA concentration of 0–60 μM were added, and followed by 0.1 M NaCl sodium phosphate buffer to volume. The sample solutions thus obtained were 20 μM TFZ-0.1 M NaCl sodium phosphate buffer solutions containing 0 to 60 μM HSA, which bound LCFA at an LCFA/HSA molar ratio of 0 to 7. The reference solutions were prepared in the same manner as the sample solutions without the drug. Each flask was shaken for a short time and incubated at 37°C for 30 min before the spectral measurement.

### Measurement of Absorption and Second-Derivative Spectra

The absorption spectra were recorded for the wavelength region of 230 to 310 nm in a 1-cm cuvette with a slit width of 2 nm and a wavelength interval of 0.1 nm at 37°C using a double-beam spectrophotometer (Hitachi U-3210) equipped with a thermostatic cell holder. Second-derivative spectra were calculated from the absorption spectra using a Visual Basic program based on the Savitzky-Golay method employing a cubic polynomial 17-point convolution and a wavelength interval (Δλ) of 0.6 nm [8, 9].

### Circular Dichroism (CD) Measurement

The CD sample solutions of 200 μM HSA containing LCFA at various LCFA/HSA molar ratios were prepared in 0.1 M NaCl buffer solution using a method similar to that described in “Preparation of Sample Solutions.” The CD spectra were recorded for the wavelength region of 250 to 300 nm in a 1-cm cuvette with a slit width of 1.0 nm and a wavelength interval of 0.2 nm at 37°C using a circular dichroism spectrometer (JASCO J-720WI) equipped with a thermostatic cell holder.

### Data Analysis

When the effect of residual background signals is entirely eliminated in the second-derivative spectra of the sample solutions, the derivative intensity difference (Δ*D*) measured at a specific wavelength before and after the addition of HSA is proportional to the concentration of TFZ bound to HSA [8, 9]. Therefore, the fraction of TFZ bound to HSA, *α*, can be given by:

Eq. 1 $$\alpha =\Delta D/\Delta D_{\text{max}}$$

where Δ*D*_max_ represents the Δ*D* when all of the TFZ molecules in the sample solution are bound to HSA [8, 9]. Thus, with the total TFZ concentration in the sample solution ([*C*_t_]), the molar concentrations of TFZ bound to HSA ([*C*_b_]) and unbound in the water phase ([*C*_u_]) are given by *α*[*C*_t_] and (1 − *α*)[*C*_t_], respectively. Also, the number of moles of TFZ bound per mole of HSA, *r*, is given by *α*[*C*_t_]/[*P*], where [*P*] represents the concentration of HSA.

With these values, the binding of TFZ with HSA containing LCFA was examined by the Scatchard equation.

### Calculation of the K Value

For the partition-like nonspecific binding, the binding constant *K* is given as in Eq. 2 [20].

Eq. 2 $$\frac{r}{\left[C_{\text{u}}\right]}=\frac{\left[C_{\text{b}}\right]}{\left[P][C_{\text{u}}\right]}=K$$

Using Δ*D, K* can be written as follows [8, 9]:

Eq. 3 $$K=\frac{\left(\Delta D/\Delta D_{\text{max}}\right)}{\left(1-\Delta D/\Delta D_{\text{max}})[P\right]}$$

Equation 3 can be transformed to Eq. 4:

Eq. 4 $$\Delta D=\frac{K[P]\Delta D_{\text{max}}}{\left(1+K[P]\right)}$$

The *K* values were calculated by fitting the observed data of the Δ*D* values and the HSA concentration [*P*] to Eq. 4 using a nonlinear least-squares method [8, 9]. In the calculation, the Δ*D*_max_ value was also treated as a parameter for which the initial value was obtained by extrapolating the value of [*P*] to ∞ in the plot of 1/Δ*D* versus 1/[*P*].

## Results and Discussion

### Absorption Spectra

**Fig. 2:**
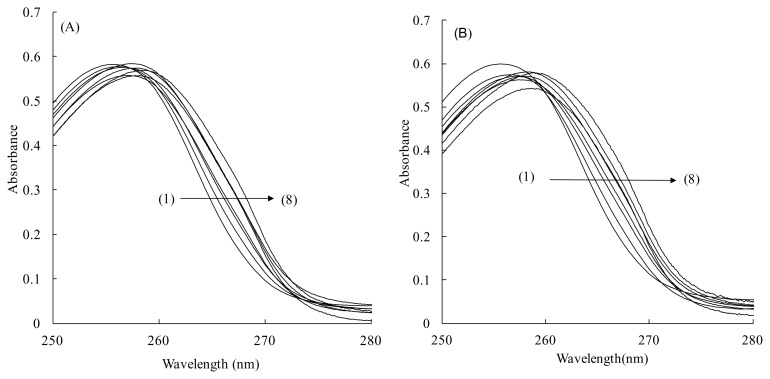
Absorption spectra of 20 μM TFZ in buffer solutions (pH 7.40, NaCl 0.1 M) in the presence of several amounts of HSA containing LCFA. HSA concentration (μM): (1) 0 – (8) 64. LCFA (LCFA/HSA molar ratio): (A) CPA (1), (B) OLA (5).

The absorption spectra of 20 μM TFZ in 0.10 M NaCl buffer solutions in the presence of various amounts of HSA containing LCFA at a certain LCFA/HSA molar ratio are depicted in Fig. 2(A) for CPA and (B) for OLA, as typical results of spectrophotometric titration experiments. The absorption maxima of TFZ in both Fig. 2(A) and (B) show bathochromic shifts according to the increase in the concentration of HSA. However, it is evident that the counterbalance of the background signals of HSA in the sample and reference beams is incomplete, even though the solutions in the sample and reference cuvettes were prepared carefully to contain the same amount of HSA. Neither the results in Fig. 2(A) nor those in (B) exhibit a distinct isosbestic point. All of the other spectra obtained with the other LCFAs showed similar results. When the background signal level was very high, it was usually difficult to cancel the background signal effects completely to obtain a flat and zero-level baseline. Thus, it was difficult to obtain further spectral data for calculating the binding constants from these absorption spectra. To overcome these defects in absorption spectra, their second-derivative spectra were acquired.

### Second-Derivative Spectra

**Fig. 3:**
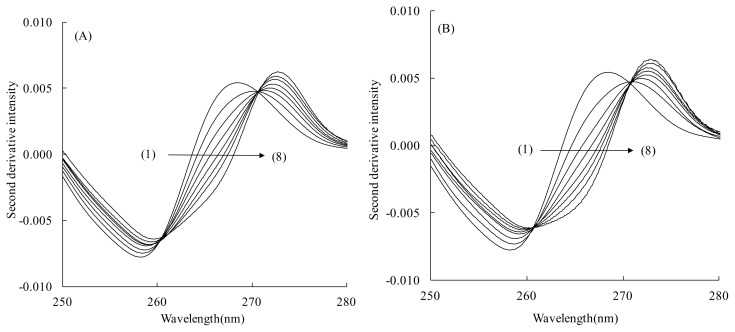
Second-derivative spectra of TFZ calculated from the absorption spectra of Fig. 2(A) and (B), respectively.

The second-derivative spectra obtained from the absorption spectra in Fig. 2(A) and (B) are depicted in Fig. 3(A) and (B), respectively. Figures 3(A) and (B) show a bathochromic shift with an increase in the HSA concentration similarly to the absorption spectra in Fig. 2(A) and (B). Furthermore, Fig. 3(A) and (B) clearly show the distinct derivative isosbestic points, which indicate that the residual background signal effects were entirely eliminated in the second-derivative spectra, and also confirm that TFZ exists in two states that exhibit different derivative spectra, i.e., a free state in the buffer phase as well as an HSA-bound state [21]. All of the second-derivative spectra for the other LCFA/HSA ratios of the four LCFAs gave similar results. These facts confirmed that the spectral data for calculating the *K* value of TFZ for HSA-containing LCFA can be obtained from the second-derivative spectra.

### Scatchard Plot

**Fig. 4:**
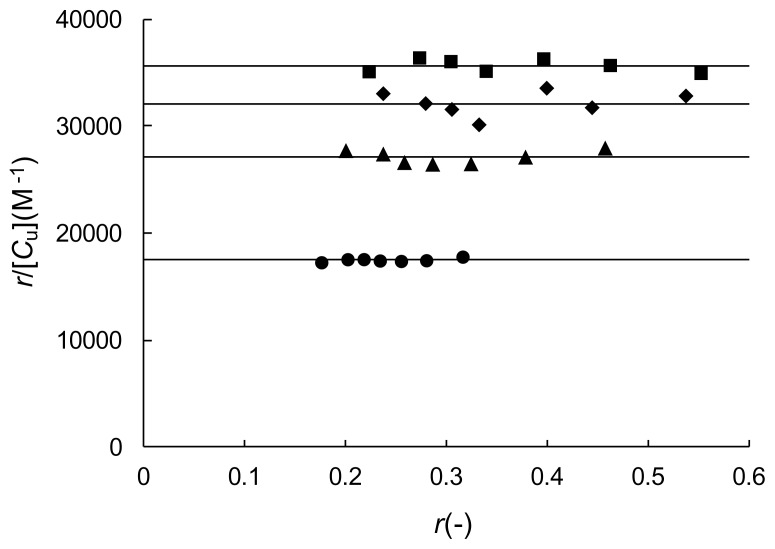
Scatchard plot for the binding of TFZ to HSA containing LCFA. LCFA (LCFA/HSA molar ratio): CPA (1) (●), LRA (4) (■), OLA (5) (▲), LNA (2) (◆).

The Δ*D* value at several HSA concentrations in the second-derivative spectra was measured at 265 nm. In principle, the Δ*D* value measured at any wavelength can be used for the calculation of the Scatchard parameters. However, from the viewpoint of the accuracy and reproducibility of the calculation results, large Δ*D* values are preferable. Therefore, judging from the spectra in Fig. 3, and referring to the previous BSA studies [8, 9], the Δ*D* value was measured at 265 nm. We also examined the Δ*D* value with two other wavelengths, 267 and 258 nm, and confirmed that the Δ*D* value at 265 nm gave the best results. The Δ*D*_max_ value was obtained by the extrapolation of the reciprocal plots of the Δ*D* value and the HSA concentration, [*P*]. Using the obtained Δ*D*_max_ value, the *α* value was calculated according to Eq. 1. Then, the *α* value was used to calculate the *r* and *r*/*C*_u_ values and the Scatchard plot analysis was performed. Typical results for each LCFA are depicted in Fig. 4. Each plot shows a straight line parallel to the abscissa. All of the Scatchard plots for the other LCFA/HSA molar ratios of each of the four LCFAs showed results similar to those presented in Fig. 4.

It has been reported that for the partition-like nonspecific binding, the Scatchard plot will yield a straight line parallel to the abscissa [20]. The above results from the Scatchard analysis indicate that the nonspecific binding model can be safely adopted to account for the interactions of TFZ with HSA containing the LCFAs. As described above, our previous ^19^F NMR results [7] revealed that in the presence of 0.1 M Cl^−^ the fraction of TFZ specifically bound to Site II on HSA was very small due to binding competition with Cl^−^. Therefore, under the conditions of this study, i.e., in the presence of a physiological concentration of 0.1 M Cl^−^, TFZ binding to HSA can be treated as a partition-like nonspecific binding.

### Calculation of the K Value

The binding constant (*K*) at several LCFA/HSA molar ratios was calculated based on Eq. 4 with a nonlinear least-squares method using the experimental data of HSA concentration, [*P*], and the Δ*D* values. For each LCFA/HSA molar ratio, three independent experiments were performed and all of the *K* values were obtained with good reproducibility. The results are summarized in Table 1, and intriguingly reveal that LCFA has a positive or negative effect on the binding of TFZ to HSA depending on its chain length and the LCFA/HSA molar ratio.

**Fig. 5:**
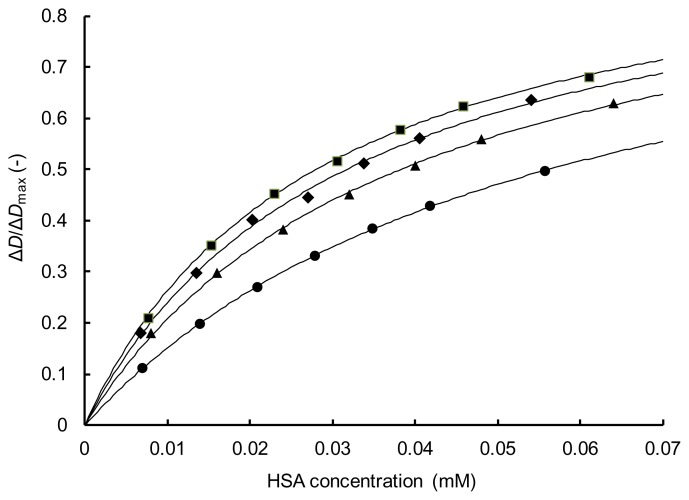
Fraction of TFZ bound to HSA containing LCFA. LCFA (LCFA/HSA molar ratio): CPA (1) (●), LRA (4) (■), OLA (5) (▲), LNA (2) (◆).

To confirm the accuracy of the obtained *K* values, the fraction of TFZ bound to HSA (*α*) was calculated from Eq. 1 using the obtained *K* and Δ*D*_max_ values. In Fig. 5, typical results are shown for each of the four LCFAs as a plot of *α* vs. the HSA concentration. This plot was made by using the same data as used for the Scatchard plot in Fig. 4. All of the plotted experimental values fell close to the calculated curves, thus demonstrating the reliability of the obtained *K* values. Similar results were obtained for the other LCFA/HSA molar ratios of the four LCFAs.

### Effect of LCFA on the K Value

**Fig. 6:**
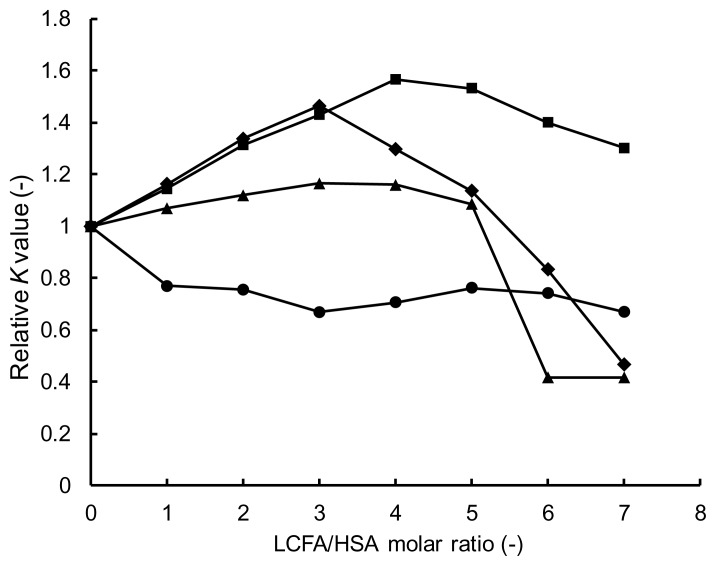
Effect of LCFA on *K* value. LCFA: CPA (●), LRA (■), OLA (▲), LNA (◆).

To compare the effect of each LCFA on the binding of TFZ to HSA, the *K* values listed in Table 1 were divided by the *K* value obtained for HSA not containing LCFA, and the calculated relative *K* values were plotted as a function of the LCFA/HSA molar ratio in Fig. 6 for CPA, LRA, OLA, and LNA, respectively. Interestingly, the results revealed that the effects of LRA (12:0), OLA (18:1), and LNA (18:2) showed a similar trend of *K* values that increased with increasing LCFA/HSA molar ratio from 1 to 3 or 4, and decreasing *K* values for larger ratios. The most enhancing effect on the TFZ binding was given by LRA, which has a saturated chain of 12 carbons, i.e., when the LRA/HSA molar ratio was 4, the *K* value was enhanced to more than 1.5-fold compared to the value for HSA alone. In the case of LCFAs having unsaturated bonds, OLA, and LNA, when HSA contained more than five molecules of these LCFAs per HSA molecule, the *K* value decreased to 40% of the value for HSA alone. On the other hand, CPA did not have an enhancing effect on the *K* value at any CPA/HSA ratio, and in fact tended to decrease the TFZ binding to HSA regardless of its content in HSA, i.e., the *K* value for HSA containing CPA was about 80% of the value for HSA not containing LCFA. From these results, it was clearly confirmed that LCFAs influence the binding of TFZ to HSA depending on their content in HSA and on their chain-length. However, because several kinds of LCFAs are bound to HSA in the blood, the influence of the LCFA binding on the TFZ-HSA binding is considered to be dependent on its content, i.e., it enhances the binding of TFZ at an LCFA/HSA ratio of 1 to 4, while further increases in the content weaken the TFZ binding. As mentioned above, the LCFA content varies with the physical condition of the body, and thus it has been suggested that the concentration of free TFZ in the blood may depend on the physical condition of the body, e.g., the length of time since the last meal, the presence of diabetes, etc.

Fatty acid effects on drug-HSA binding that are dependent on the number of fatty acid molecules present in HSA have also been reported for warfarin [22]. In our preliminary experiments with BSA, OA showed effects similar to other phenothiazine drugs, such as chlorpromazine, prochlorpromazine, and trifluperazine (data not shown). Therefore, it can be considered that the results obtained from the present study are not confined to TFZ-HSA binding as a special case.

### Circular Dichroism (CD) Spectra

**Fig. 7:**
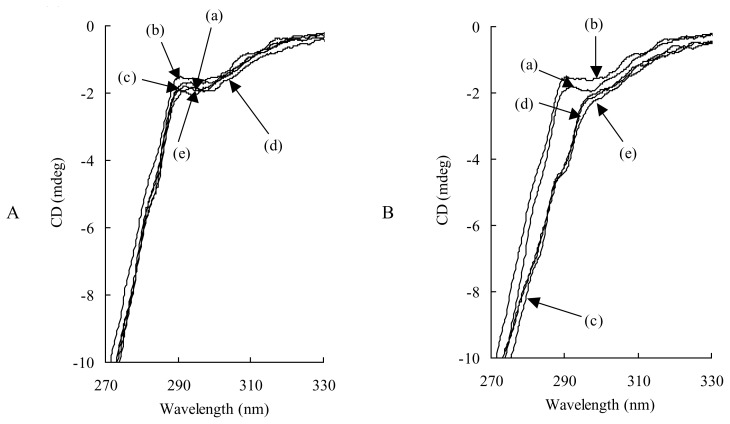
CD spectra of 200 μM HSA containing CPA (A) and OLA (B) at several molar ratios in a 0.1 M NaCl buffer solution. (A) (a) HSA in buffer solution (without NaCl). CPA/HSA molar ratio: (b) 0, (c) 1, (d) 3, (e) 7. (B) (a) HSA in buffer solution (without NaCl). OLA/HSA molar ratio: (b) 0, (c) 1, (d) 2, (e) 7.

To see whether these LCFA effects are dependent on the structural change of HSA induced by binding with each LCFA, the CD spectra of 200 μM HSA containing LCFA at 0–7 LCFA/HSA molar ratios were measured in a 0.1 M NaCl buffer solution. The results for CPA and OLA are depicted in Fig. 7 as typical samples. As seen in Fig. 7(A), CPA did not cause any change in the CD spectrum of HSA at any content examined. On the other hand, as shown in Fig. 7(B), the binding of OLA to HSA caused a small spectral change around the wavelength of 290 nm, suggesting that the structural change of HSA was related to tryptophan residues. Quite similar CD results were obtained for LRA and LNA (data not shown). However, in all of the CD spectra, spectral change was induced even at an LCFA:HSA molar ratio of 1:1, and further increases in the LCFA content did not induce further spectral changes, despite the fact that the *K* value was influenced by LCFA content in HSA. Therefore, to account for the results that the LCFA binding to HSA increased or decreased the binding constant of TFZ to HSA depending on the kind of LCFA and its content in HSA, further investigations, e.g., thermodynamic and/or crystallographic approaches, will be necessary [23, 24].

## Conclusion

By employing a second-derivative spectrophotometric method, the effect of LCFAs (CPA, LRA, OLA, and LNA) on the binding of TFZ to HSA was examined in a 0.1 M NaCl buffer solution. Using the derivative intensity change induced by the addition of HSA, a Scatchard plot analysis was performed and the results showed that the binding of TFZ to HSA containing LCFA could be analyzed as a partition-like nonspecific binding. The binding constant, *K*, showed that for LRA, OLA, and LNA, the binding increased according to the increase of LCFA contents in HSA up to the LCFA/HSA molar ratio of 3 or 4. However, at higher ratios, the *K* value decreased. For OLA or LNA, at the LCFA/HSA ratio of 6–7 the *K* value decreased to 40% of the value for HSA alone. CPA, having the shortest chain-length (8 carbons) in the LCFAs examined, did not induce any increase in the *K* value, but rather induced a 20% decrease in the *K* value regardless of its content in HSA.

As an HSA molecule binds up to seven molecules of LCFA according to the physical condition of the body, e.g. ordinarily the LCFA content of HSA is 1–2, but during a fast or after hard exercise, or in subjects suffering from diabetes, it binds LCFA at the highest level of 6–7, the results of the present study suggest that the concentration of unbound TFZ in the blood, which relates closely to its pharmacological activity, may be influenced by the condition of the body through the LCFA content in HSA. Therefore, the results of the present study are pharmacokinetically, pharmacologically, and clinically very important.

## Figures and Tables

**Tab. 1:** Binding constant of TFZ to HSA containing LCFA at several LCFA/HSA molar ratios.

LCFA/HSA (−)	a*K*×10^−4^ (M^−1^)			

	CPA	LRA	OLA	LNA
0	2.33±0.14	2.33±0.14	2.33±0.14	2.33±0.14
1	1.79±0.03	2.66±0.22	2.50±0.16	2.71±0.17
2	1.76±0.06	3.06±0.12	2.61±0.25	3.11±0.03
3	1.56±0.13	3.33±0.20	2.90±0.08	3.41±0.05
4	1.65±0.14	3.65±0.11	2.70±0.06	3.02±0.08
5	1.48±0.16	3.57±0.05	2.53±0.11	2.65±0.21
6	1.73±0.15	3.26±0.27	0.97±0.06	1.94±0.13
7	1.56±0.09	3.03±0.25	0.96±0.06	1.10±0.04

a mean ± standard deviation (n = 3).
